# Cutting State Diagnosis for Shearer through the Vibration of Rocker Transmission Part with an Improved Probabilistic Neural Network

**DOI:** 10.3390/s16040479

**Published:** 2016-04-06

**Authors:** Lei Si, Zhongbin Wang, Xinhua Liu, Chao Tan, Lin Zhang

**Affiliations:** 1 School of Mechatronic Engineering, China University of Mining & Technology, No. 1 Daxue Road, Xuzhou 221116, China; lei.si@cumt.edu.cn (L.S.); l_xinhua_2006@126.com (X.L.); tccadcumt@126.com (C.T.); lin.zhang_2014@hotmail.com (L.Z.); 2 School of Information and Electrical Engineering, China University of Mining & Technology, No. 1 Daxue Road, Xuzhou 221116, China

**Keywords:** shearer cutting state diagnosis, probabilistic neural network, fruit fly optimization algorithm, Kullback–Leibler divergence, distance-based evaluation, feature extraction

## Abstract

In order to achieve more accurate and reliable identification of shearer cutting state, this paper employs the vibration of rocker transmission part and proposes a diagnosis method based on a probabilistic neural network (PNN) and fruit fly optimization algorithm (FOA). The original FOA is modified with a multi-swarm strategy to enhance the search performance and the modified FOA is utilized to optimize the smoothing parameters of the PNN. The vibration signals of rocker transmission part are decomposed by the ensemble empirical mode decomposition and the Kullback-Leibler divergence is used to choose several appropriate components. Forty-five features are extracted to estimate the decomposed components and original signal, and the distance-based evaluation approach is employed to select a subset of state-sensitive features by removing the irrelevant features. Finally, the effectiveness of the proposed method is demonstrated via the simulation studies of shearer cutting state diagnosis and the comparison results indicate that the proposed method outperforms the competing methods in terms of diagnosis accuracy.

## 1. Introduction

With the rapid development of China’s economy, the demand for coal is still ongoing and the efficiency and safety of coal production is becoming more and more noticeable in the coalmining industry. However, in some mines, the coalmining environment is extremely harsh and the automatization level of coalmining machines is rather low. The operators cannot accurately estimate the cutting state only depending on their visual and auditory information. This phenomenon will lead to some problems, such as poor coal quality and low mining efficiency. Moreover, many safety accidents in collieries occur frequently. As the key equipment in coalmining face, shearer plays an important role in the process of coal production. Diagnosing its cutting state, which aims at whether the shearer is cutting coal or rock, is an indispensable precondition for improving the automatization degree.

In the past decades, some studies have focused on the coal–rock identification technology to roughly estimate the cutting state of shearer and many kinds of coal–rock recognition methods have been proposed successively. The most representative methods mainly consist of γ-ray detection means [1], radar detection means [2,3], acoustic detection means [4], infrared detection means [5], image detection means [6,7], and so on [8,9,10]. Although many coal-rock recognition methods have been developed, they have some common disadvantages. Firstly, the coal-rock detectors in the above literature are complex and require severe geological conditions of coal seam, which cannot satisfy extensive application during practical production. Furthermore, the recognition rate is sensitively influenced in the conditions of gangue included in the coal seam.

In recent years, due to the inevitable shortcomings mentioned above, the diagnosis methods for shearer cutting state based on coal-rock recognition have gradually left using our field of vision. Considering the fault diagnosis methods for other traditional machines, it is known that sensors can tackle the problem of perception by providing information of machines, and vibration signal analysis is becoming the most commonly used method and also proved to be efficient in various real applications [11,12]. For a shearer, the rocker is the critical component and can transmit the power from cutting motor to the cutting drum. When the shearer is working in different cutting conditions, the state information of rocker transmission part will change remarkably and using the vibration of this part can comprehensively reflect the cutting state of shearer.

The process of state detection is essentially a process of pattern recognition and classification. Several techniques have been successfully used for classification problems, such as neural network (NN) [13,14], support vector machine [15], naive Bayes [16], and so on. The Neural Network, first proposed by Rosenblatt in the late 1950s [17], is one of the most well-known and widely used techniques for classification. Since that time, many NN models have been derived and developed, including back propagation networks, radial basis function networks, multilayer perceptron networks, feedback networks and probabilistic neural networks (PNNs). These models differ from each other in terms of architecture, behavior and learning approaches, hence they are suitable for solving different problems such as series forecasting [18,19,20], weather prediction [21], fault diagnosis [22] and pattern recognition [23].

PNN is an effective data classifier proposed in [24,25], and has been widely used for classification tasks in several fields of science. In the training process, PNN solely requires a single input-output signal pass to compute its response. However, the correctness of the model response is obviously affected by an appropriate choice of the smoothing parameter (σ in short). With the development of evolutionary algorithms [26,27], diverse procedures have been developed to solve this problem, such as genetic algorithm (GA) [28], particle swarm optimization (PSO) algorithm [29], reinforcement learning algorithm [30], and conjugate gradient descent [31]. On the basis of the above methods, this paper introduces a new approach for adaptive computation of the smoothing parameter of the PNN model. This method is based on an evolutionary algorithm of fruit fly optimization algorithm (FOA), which is coupled with a multi-swarm strategy to improve the evolutionary performance of original FOA.

In addition, another key step in the process of state diagnosis is how to extract the state features and identify the condition from the signals. Some typical methods for feature extraction are Fourier transform (FT) [32,33], wavelet transform (WT) [34], Hilbert transform (HT) [35], empirical mode decomposition (EMD) and ensemble empirical mode decomposition (EEMD) [36,37,38]. These methods possess their own characteristics and application scenarios. In this work, the EEMD method is utilized to decompose the measured signals and the Kullback-Leibler divergence (KLD), also called relative entropy, is used to identify the false components from the EEMD. However, since some of the extracted features may be insensitive to some specific state modes, the distance-based evaluation approach (DE) [39] is employed to remove the irrelevant features in this study. The feature vectors composed by these features can generate samples of the modified PNN for state diagnosis.

The remaining parts of the paper are organized as follows. Section 2 summarizes some related works about our proposed method. Section 3 describes the basic concept of PNN and presents the parameters optimization of PNN model based on modified FOA in detail. Section 4 presents the diagnosis process for shearer cutting state based on proposed method. Section 5 provides some examples and comparisons of proposed model with other methods. Section 6 summarizes the conclusions and some future works of our paper.

## 2. Related Works

### 2.1. Feature Extraction Methods

Feature extraction is the premise to realize pattern recognition and state diagnosis and many advanced signal processing algorithms have been proposed to extract a set of features reflecting various types. In [32], the windowed Fourier transform method is used to study the thermodiffusion phenomenon to improve the contrast of the reconstructed images and reduce the noise. Veer *et al.* [33] employed the short-time Fourier transform and wavelet transform to extracted features of reordered signals for the recognition of arm movements and the compared results indicated that wavelet was a more useful and powerful tool for analyzing signals. In [34], a modified threshold denoising method based on wavelet transform was adopted to improve the quality of a signal polluted by noises. Yu *et al.* [40] proposed a novel feature extraction method for frequency bands to select salient features from the marginal spectrum of vibration signals by Hilbert-Huang Transform. In [41], a parallel EEMD algorithm based on Map Reduce model was designed to improve the computational efficiency and an improved envelope reconstruction algorithm was proposed to reduce the envelope error. Demir *et al.* [42] presented the utilization of EMD in hyperspectral images to increase the classification accuracy using support vector machine-based classification. In [43], the EEMD together with the Hilbert transform was employed to extract the time- and frequency-domain features, and a new data-driven fault diagnosis method was proposed by the integration of kernel density estimation and Kullback-Leibler divergence.

### 2.2. Optimization and Improvement of PNN

PNN is a well-known and efficient approach for classification and some improved strategies have been integrated with PNN to find high quality solutions (with respect to classification accuracy). For example, in [28], a single smoothing parameter for the whole PNN was identified using a GA to obtain satisfactory classification accuracy. In [29], an evolutionary PNN was proposed based on PNN and PSO, and the PSO was used to optimize the matrix of smoothing parameters for each class of neurons. Kusy *et al.* [30] proposed new methods for the choice and adaptation of the smoothing parameter of the PNN. These methods are based on three reinforcement learning algorithms: *Q*(0)-learning, *Q*(λ)-learning and stateless Q-learning. In [44], the authors proposed a method that hybridized the firefly algorithm with simulated annealing (denoted as SFA) to optimize the weights of the standard PNN model. Chtioui *et al.* [45] developed a two-step numerical procedure for the optimization of the smoothing parameters of PNN: a rough optimization by the conjugate gradient method and a fine optimization by the approximate Newton method. In [46], the *Q*(0)-learning algorithm was utilized for the adaptation of PNN smoothing parameter with four types of parameter structures.

### 2.3. Discussion

According to the scientific contributions concerned with feature extraction methods and PNN, we find that the number of studies about the application of FOA to the smoothing parameter computation for PNN model is still small, and there is a lack of studies where different types of data-driven diagnosis methods are thoroughly applied in the field of shearer cutting state. With the above literature in mind, we develop a new diagnosis method for shearer cutting state through the vibration of rocker transmission part. The smoothing parameters of PNN are optimized for each input attribute using a modified FOA. The KLD and DE methods are employed to select effective features of original signal and the signals preprocessed by the EEMD. Some simulation studies are carried out to verify the feasibility and superiority of proposed method.

## 3. Probabilistic Neural Network and Parameter Optimization

### 3.1. Probabilistic Neural Network

**Figure 1:**
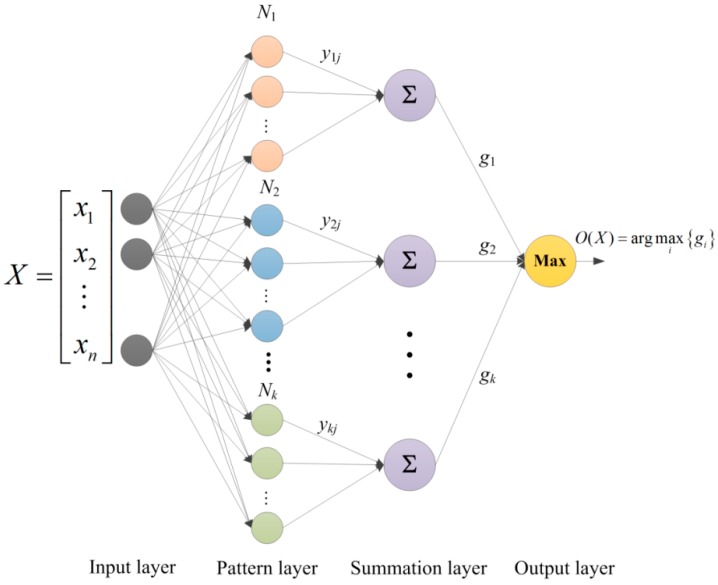
The structural diagram of probability neural network.

Probabilistic neural network (PNN) is developed based on the Bayes classification rules and the probability density function estimation method of Parzen window. PNN is a supervised neural network that is widely used in the area of pattern recognition and it has infinite potential in fault diagnosis for its parallel-distributed processing, self-organization and self-study ability. A PNN consists of four layers: input, pattern, summation and output, as illustrated in Figure 1.

The input layer is the first layer of neurons. Each input neuron represents a separate attribute in the training/testing datasets (for example, from *x*_1_ to *x_n_*). The number of neurons in input layer is equal to the number of attributes in the dataset. The second layer contains *m* neurons, which is equal to the number of the training samples of all classes *k*, that is $m=N_{1}+N_{2}+\cdots +N_{k}$. The Euclidean distances between the input sample and training samples are calculated to acquire the similarity according to the Gaussian probability density function:

(1) $$y_{ij}=\exp\left(-\frac{{\left\|X-X_{j}^{\left(i\right)}\right\|}^{2}}{2\sigma^{2}}\right)\;i=1,2,\cdots ,k$$

where $X=[x_{1},x_{2},\cdots ,x_{n}]$ is the argument vector to be classified, $X_{j}^{\left(i\right)}=[x_{j1}^{\left(i\right)},x_{j2}^{\left(i\right)},\cdots ,x_{jn}^{\left(i\right)}]$ is the *j*th training vector (*j* = 1, ..., *N_i_*) from the *i*th class, *N_i_* is the number of the training samples of the *i*th class, and *σ* denotes the smoothing parameter.

The third layer contains summation units needed to complete the probability estimation. There are as many summation units as classes. Each summation unit receives input data only from those pattern units belonging to its respective class. Using the Parzen method, the probability density function for multiple variables can then be expressed as follows:

(2) $$g_{i}=\frac{1}{{\left(\sigma \sqrt{2\pi }\right)}^{n}N_{i}}\sum_{j=1}^{N_{i}}y_{ij}\;i=1,2,\cdots ,k$$

Finally, the output layer can determine the decision category for input vector *X*. In this layer, the maximum of the summation node outputs can be found. Through a simple comparison, shown as Equation (3), only the unit corresponding to the class with the highest summation unit value produces an output of one, while others generate a value of zero thereby indicating the classification decision for the input vector.

(3) $$O(X)=\arg\;\max\left\{g_{1},g_{2},\cdots ,g_{k}\right\}$$

In the training process of PNN, the most important aspect is the selection of smoothing parameter σ. A proper choice of this parameter has a major impact on the classification ability of the network. Traditionally, the PNN only need to set the same smoothing parameter based on the experience, which cannot fully reflect the correlation degree between samples. In this paper, the smoothing parameter is computed separately for each attribute of the samples. This type of model is a more elastic classifier, since in such a case, the influence of each variable on neighboring points differs. Then, the *i*th summation neuron provides the following output:

(4) $$g_{i}=\frac{1}{{\left(\sqrt{2\pi }\right)}^{n}N_{i}\prod_{l=1}^{n}\sigma_{l}}\sum_{j=1}^{N_{i}}\exp\left(-\frac{{\left\|X-X_{j}^{\left(i\right)}\right\|}^{2}}{2\sigma_{l}^{2}}\right)\;i=1,2,\cdots ,k$$

As a new type of swarm intelligence method, fruit fly optimization algorithm is used to optimize the smoothing parameters (the total number of the parameters is equal to *n*), in order to achieve the optimal classification effect. The specific optimization process will be provided in the following parts.

### 3.2. Modified Fruit Fly Optimization Algorithm

The conventional fruit fly optimization algorithm (FOA) was proposed by Pan [47] and belongs to a kind of interactive evolutionary computation method. It can find global optimization based on the food finding behavior of the fruit fly. According to the food finding characteristics of fruit fly swarm, the conventional FOA procedure can be shown in literature [47].

From the FOA procedure, it has some disadvantages that limit its performance. The search mechanism for optimal solution reveals that it does not have high probability of mutation. When some swarms meet the optimum solution, another swarm will follow that solution. Hence, fruit fly swarm loses its ability to search for a global optimum solution, which leads to trap in local optimum and reduce the convergence speed and convergence accuracy, namely the problem of premature convergence. In order to overcome the aforementioned disadvantages, this paper puts forward the multi-swarm strategy for original FOA, which refers to modified FOA or MFOA. In this paper, the fruit fly swarm is split into several sub-swarms and each sub-swarm searches the optimal solution independently and simultaneously. This method uses several sub-swarms mainly in order to enhance the diversity of solutions and achieve an effective exploration to avoid local optimal or premature. In this paper, the fruit fly swarm is divided into three sub-swarms equally. The first group is assigned to find a new search space with wide area, the second group is assigned to find nearby optimum space, and the last group is assigned to find the optimal solution in a change search space.

The implement procedure of the proposed MFOA is summarized as follows.

Step 1: Initialize the swarm location range (*LR*), maximum iteration number (*Maxgen*), population size (*sizepop*). Randomly generate the initial fruit fly swarm location (*X_axis*, *Y_axis*).

(5) X_axis=rand(LR),Y_axis=rand(LR)

Step 2: Generate the random direction and distance for the *i*th fruit fly. Each sub-swarm is conducted independently as follows:

For sub-swarm 1,

(6) $$\begin{array}{l} X_{i}=random\;value\\ Y_{i}=random\;value \end{array}$$

For sub-swarm 2,

(7) $$\begin{array}{l} X_{i}=X\_axis+random\;value\\ Y_{i}=Y\_axis+random\;value \end{array}$$

For sub-swarm 3, the random direction and distance can be generated as follows:

(8) $$\begin{array}{l} X_{i}=X\_axis+\eta (t)\times random\;value\\ Y_{i}=Y\_axis+\eta (t)\times random\;value\\ \eta (t)=\left(b-a\right)\times {\left(\frac{Maxgen-t}{Maxgen}\right)}^{2} \end{array}$$

where [*a*, *b*] denotes the flight distance range of fruit fly; and η(*t*) denotes the adjustment factor and is set according to the iteration times t. In earlier iterations, big η(*t*) value may increase the diversity of solution vectors for global exploration, while in later iterations small η(*t*) value may enhance the fine-tuning of solution vectors by local exploitation.

Step 3: Estimate the distance to the origin (*Dist*) and calculate the smell concentration judgment value (*S*).

(9) $$Dist_{i}=\sqrt{X_{i}^{2}+Y_{i}^{2}},S_{i}=1/Dist_{i}$$

Step 4: Substitute smell concentration judgment value (*S*) into smell concentration judgment function (or called fitness function) so as to find the smell concentration (*Smell_i_*) of the individual location of the fruit fly.

(10) $$Smell_{i}=Function(S_{i})$$

Step 5: Find out the fruit fly with best smell concentration among the *j*th sub-swarm.

(11) $$\left[bestSmell_{j}\;bestIndex_{j}]=Best(Smell\right)$$

Step 6: Keep the best smell concentration value and *x*, *y* coordinate among each sub-swarm. At this moment, each sub-swarm will use vision to fly independently towards that location.

(12) $$\begin{array}{l} Smellbest_{j}=bestSmell_{j}\\ X\_axis_{j}=X(bestIndex_{j})\\ Y\_axis_{j}=Y(bestIndex_{j}) \end{array}$$

Step 7: The global fitness *Smellbest* is set as the optimal *Smellbest_j_* and the best positions *X_axis*, *Y_axis* are set as *X_axis_j_* and *Y_axis_j_*.

Step 8: If *t* ≥ *Maxgen*, then the circulation stops; otherwise, go to Step 2.

### 3.3. Parameters Optimization for PNN Using MFOA

In this subsection, the proposed MFOA is utilized to optimize the smoothing parameters of PNN, which can be named as MFOA-FNN, in order to achieve the optimal classification effect. As the number of smoothing parameters is equal to the dimensionality of the samples, the fruit fly swarm location is set as *X_axis* = rands(1, *n*) and *Y_axis* = rands(1, *n*). In the MFOA-PNN program, we employ two variables [*X*(*i*, *n*), *Y*(*i*, *n*)] to represent the flight distance for food finding of an individual fruit fly i in each sub-swarm. The distance *Dist_i_* and smell concentration judgment value *S_i_* of the *i*th fruit fly can be calculated as follows:

(13) $$\left\{ \begin{array}{l} D(i,j)=\sqrt{X{\left(i,j\right)}^{2}+Y{\left(i,j\right)}^{2}}\\ S(i,j)=\frac{1}{D(i,j)} \end{array}\right.\;j=1,2,\cdots ,n$$

**Figure 2:**
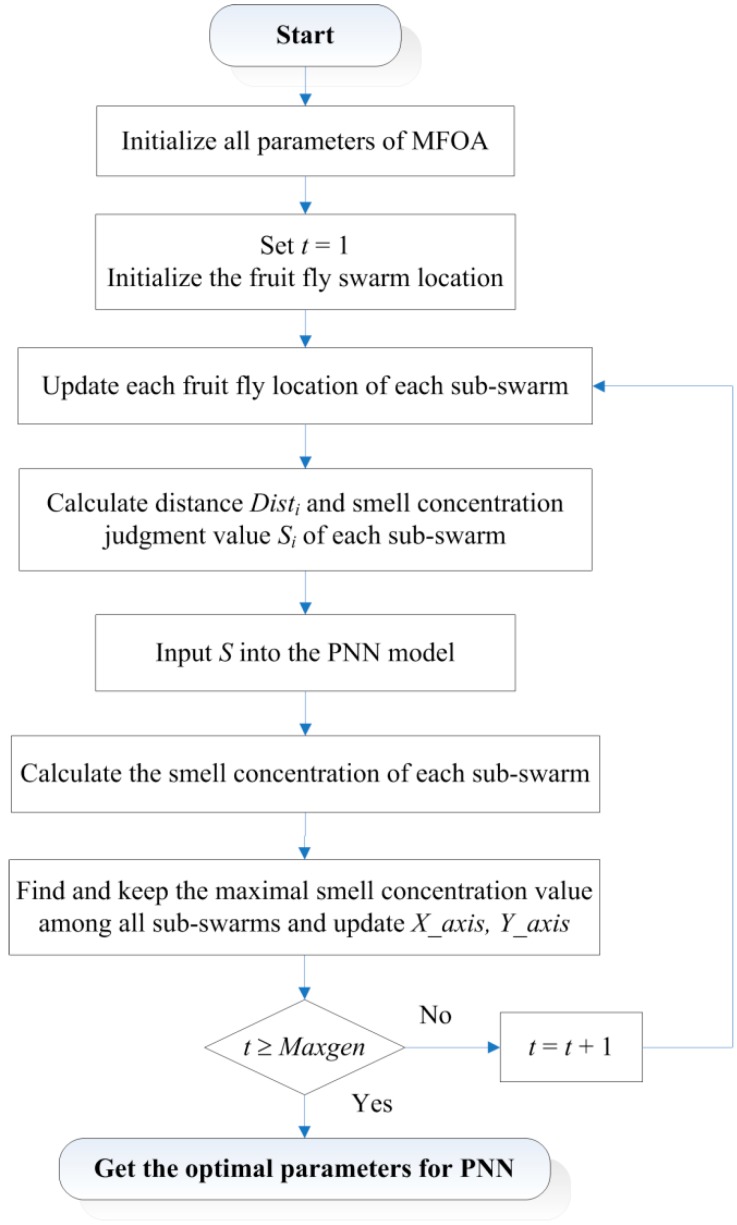
Diagram of the procedure structure of proposed model.

In the proposed model, the smoothing parameters of PNN can be represented by *S*(*i*, *j*). Then, the smell concentration *Smell_i_* (also called the fitness value of fruit fly *i*) should be calculated. We adopt the classification accuracy as the fitness function to represent the classification performance of the MFOA-PNN model. The fruit flies are operated and the sub-swarms are updated through Equations (6)–(8). When t reaches the max iterative number, the termination criterion satisfies and the optimal smoothing parameters of PNN model can be obtained. The procedure structure of the MFOA-PNN classification model is illustrated as Figure 2.

## 4. Diagnosis Process for Shearer Cutting State

**Figure 3:**
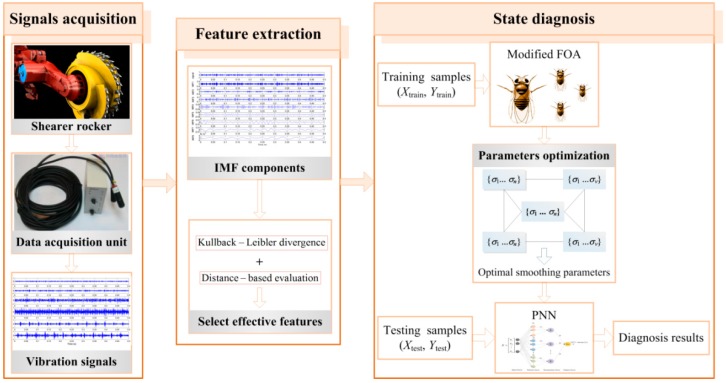
The diagnosis system for shearer cutting state based on proposed method.

The intelligent diagnosis for shearer cutting state based on proposed method is essentially a pattern recognition system, as shown in Figure 3. It mainly consists of signals acquisition, feature extraction and state diagnosis, which is explained as follows.

### 4.1. Vibration Signals Acquisition of Rocker Transmission Part

**Figure 4:**
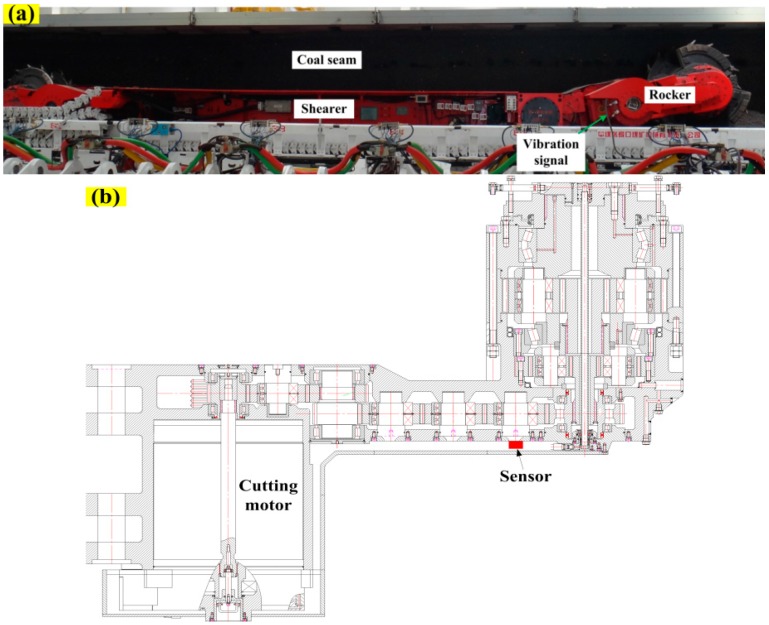
Self-designed experimental system for shearer cutting coal: (**a**) the experiment bench of shearer cutting coal; and (**b**) the installation sketch of sensor.

**Figure 5:**
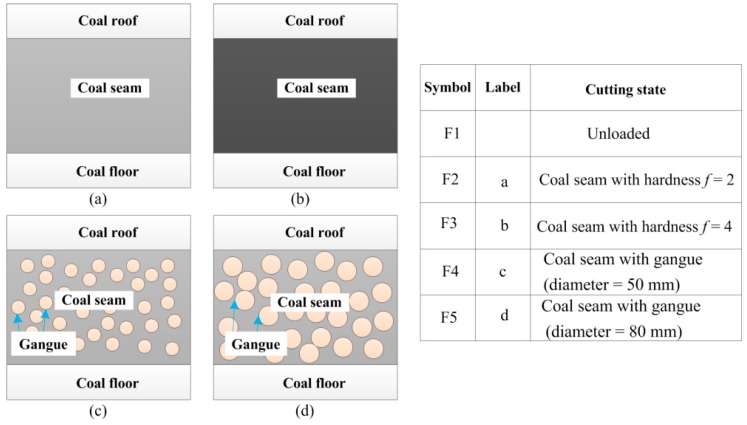
Different geological conditions of coal seam.

The cutting state diagnosis of shearer starts with data acquisition to collect the machinery working information. Vibration signal acquisition is the most commonly used method that is realized by sensors. For a shearer, the rocker transmission part delivers the power to the drum to cut the coal and rock, and its vibration can mainly reflect the shearer cutting state. However, the internal space of rocker is very narrow and the sensor can only be installed near the idlers, as shown in Figure 4b. In this study, the signals are acquired through a self-designed experimental system for shearer cutting coal, as shown in Figure 4a. In the experimental system, the coal seam was mainly divided into four parts, including two kinds of coal seams with different hardness and a coal seam with some stratums of gangue. All cutting patterns of shearer (including the shearer with unloaded condition) are represented in Figure 5.

**Figure 6:**
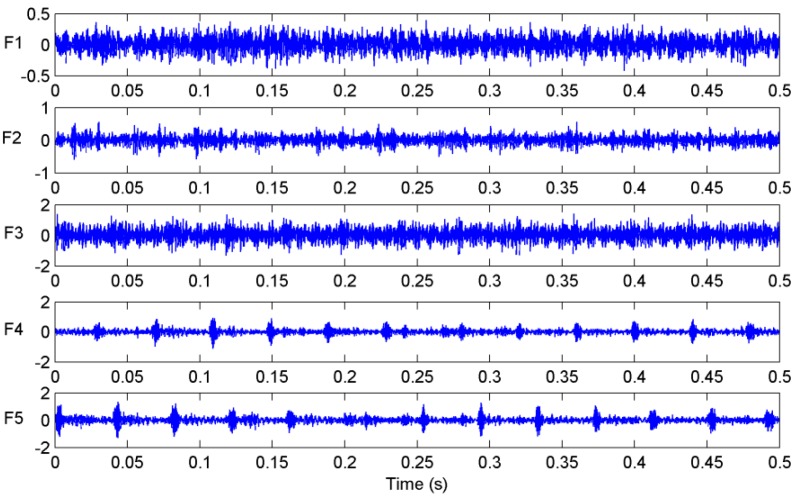
Measured vibration signals in different cutting states.

In the self-designed experimental system, a multifunctional high-speed collector performs the data acquisition and the data are collected into a notebook computer through the Universal Serial Bus interface. The sampling frequency is set as 12 kHz and the sampling time of each sample is 0.5 s. A group of measured vibration signals in different cutting states are plotted in Figure 6. Finally, we collect 200 groups with 40 groups for each cutting state to generate the samples for PNN model.

### 4.2. Feature Extraction

The feature extraction of signals is a critical initial step in any pattern recognition and fault diagnosis system. The extraction accuracy has a great influence on the final identification results. In this work, the measured vibration signals are firstly decomposed by ensemble empirical mode decomposition (EEMD) method. Then, the Kullback-Leibler divergence (KLD) is used to identify the false components from the EEMD. The decomposed signals and the original signal are estimated by nine feature parameters. Finally, the distance-based evaluation (DE) approach is used to choose some of the most effective features from the entire feature set.

#### 4.2.1. KLD-Based False Components Identification

The original signal will generate several intrinsic mode function (IMF) components after the EEMD decomposition. However, some of the IMF components, especially low frequency components, are superfluous and can be called the false components. Identifying these false components and deleting them is very meaningful to the state diagnosis of machine. In this work, the KLD method is used to measure the relation levels between the IMF components and original signal. Smaller KLD values show that the IMF components possess tighter relation with the original signal, whereas the IMF components with larger KLD values are false and should be removed. The detailed steps are listed as follows:

Step 1: The original signal *x*(*t*) is decomposed by EEMD to get *N* IMF components *c_i_*(*t*) and each *c_i_*(*t*) contains *r* data points.

Step 2: The non-parametric estimation method is utilized to compute the probability distributions of signals *x*(*t*) and *c_i_*(*t*). We assume *p*(*x*) and *q*(*x*) be two probability density functions of *x*(*t*) and *c_i_*(*t*). The following function can be defined as the kernel density estimation of *p*(*x*):

(14) $$p(x)=\frac{1}{rh}\sum_{i=1}^{r}K\left(\frac{x_{i}-x}{h}\right),\;x\in R$$

where *K*( ) is the kernel function and h is the bandwidth, which can be determined according to [48]. The most commonly used kernel function is Gaussian kernel function, namely $K(u)=\frac{1}{\sqrt{2\pi }}e^{-u^{2}/2}$. In the same way, we can get the probability density function *q_i_*(*x*) of *c_i_*(*t*).

Step 3: The following formula is defined to describe the KL distance of *p*(*x*) and *q_i_*(*x*):

(15) $$\delta (p,q_{i})=\sum p(x)\log\frac{p(x)}{q_{i}(x)}$$

The KLD value between *x*(*t*) and *c_i_*(*t*) can be calculated as follows:

(16) $$D(x,c_{i})=\delta (p,q_{i})+\delta (q_{i},p)$$

In order to facilitate the screening of effective IMF components, the KLD values are normalized as λ*_i_* through the following formula:

(17) $$\lambda_{i}=\frac{D(x,c_{i})}{\sqrt{\sum_{i=1}^{N}D{\left(x,c_{i}\right)}^{2}}}$$

In this work, the first four IMF components with smaller λ*_i_* are selected to extract features in the following subsection.

#### 4.2.2. Distance-Based Feature Selection

In order to comprehensively reflect the characteristic of signals, nine feature parameters ($f_{1}\sim f_{9}$) are defined and tabulated in Equation (18). These nine features are exacted for the original signals and the first four IMF components with smaller λ*_i_*. Thus, we can acquire 5 × 9 features in total.

(18) $$\begin{array}{c} f_{1}=\frac{1}{\sqrt{r-1}}\sqrt{\sum_{i=1}^{r}{\left(x(i)-1/r\sum_{i=1}^{r}x(i)\right)}^{2}}\;f_{2}=\frac{1}{r^{2}}{\left(\sum_{i=1}^{r}\sqrt{\left|x(i)\right|}\right)}^{2}\;f_{3}=\frac{1}{\sqrt{r}}\sqrt{\sum_{i=1}^{r}x{\left(i\right)}^{2}}\\ f_{4}=\frac{1}{(r-1)f_{1}^{3}}\sum_{i=1}^{r}{\left(x(i)-1/r\sum_{i=1}^{r}x(i)\right)}^{3}\;f_{5}=\frac{1}{(r-1)f_{1}^{4}}\sum_{i=1}^{r}{\left(x(i)-1/r\sum_{i=1}^{r}x(i)\right)}^{4}\\ f_{6}=\frac{1}{f_{3}}\max\left|x(i)\right|\;f_{7}=\frac{1}{f_{2}}\max\left|x(i)\right|\;f_{8}=\frac{r}{\sum_{i=1}^{r}\left|x(i)\right|}f_{3}\;f_{9}=\frac{r}{\sum_{i=1}^{r}\left|x(i)\right|}\max\left|x(i)\right| \end{array}$$

Nevertheless, not all the extracted features have equal contributions to fault/state diagnosis and some features are insensitive to the change of working state of machinery. In order to enhance the diagnosis accuracy and improve the computational efficiency of classification algorithms, it is necessary to delete these irrelevant features before establishing the diagnosis model. In this work, the distance-based evaluation (DE) is used to choose some of the most effective features from the entire 45 features. The main steps of DE method can be summarized as follows:

Step 1: Calculating the average distance of the *i*th feature of training samples belonging to the *j*th class (cutting state). It can be defined as follows:

(19) $$d_{j,i}=\frac{1}{N_{j}(N_{j}-1)}\sum_{\begin{array}{l} m,n=1\\ m\neq n \end{array}}^{N_{j}}\left|q_{j,i}(m)-q_{j,i}(n)\right|,i=1,2,\cdots ,Z;j=1,2,\cdots ,k$$

where *N_j_* denotes the number of samples belonging to the *j*th class; *q_j,i_*(*m*) denotes the value of the *i*th feature of the *m*th sample in the *j*th class; *Z* denotes the number of all features; *k* denotes the number of all classes (cutting states). The average distance *d_ai_* of the *i*th feature in all the *k* classes can be calculated by:

(20) $$d_{ai}=\frac{1}{k}\sum_{j=1}^{k}d_{j,i}$$

Step 2: Calculating the average value of the *i*th feature of the *N_i_* samples in the *j*th class by:

(21) $$b_{j,i}=\frac{1}{N_{i}}\sum_{m=1}^{N_{i}}q_{j,i}(m)$$

and then evaluating the average distance *d_bi_* of the *j*th classes by:

(22) $$d_{bi}=\frac{1}{k(k-1)}\sum_{\begin{array}{l} j,e=1\\ j\neq e \end{array}}^{k}\left|b_{j,i}-b_{e,i}\right|$$

Step 3: Calculating the effectiveness factor of the *i*th feature by:

(23) $$\beta_{i}=\frac{d_{bi}}{d_{ai}}$$

Step 4: Ranking all the features by the value of effectiveness factor β*_i_*. According to [33], when samples are characterized by features, a smaller distance *d_ai_* among samples within the same class is better and a bigger distance *d_bi_* between different classes is more favorable. Therefore, the features with greater effectiveness factors are preferred.

### 4.3. State Diagnosis Process

The selected features with greater effectiveness factors are used to establish the sample set for the proposed MFOA-PNN model. The samples are divided into two parts of training samples and testing samples. The training samples are used to find out the optimal smoothing parameters for PNN model based on MFOA, and the feature vectors of training samples are input to the trained PNN with optimal parameters to judge the corresponding state types of sensor signals from output results. The classification performance of proposed model is verified scientifically and reasonably.

## 5. Simulation Studies

### 5.1. Samples Preparation

**Figure 7:**
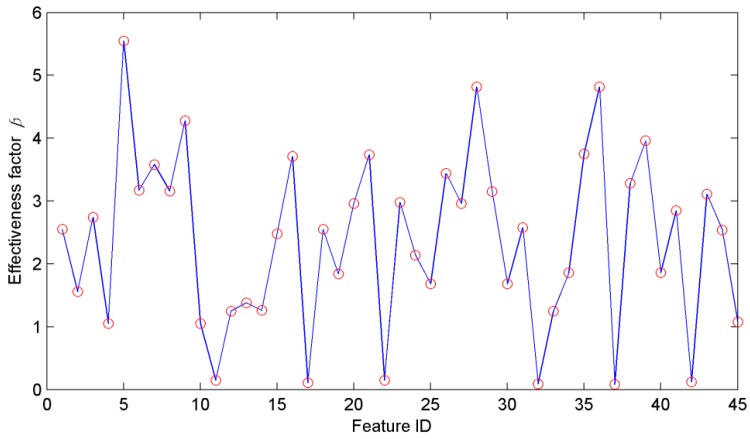
The effectiveness factor β*_i_* of all the 45 features.

In the simulation studies, the data collected from the self-designed experimental system are used to validate our proposed method and 200 groups of samples are obtained with 40 groups of samples for each cutting state. In order to determine the number of sample attributes, 45 features are first extracted from the sample set. In the EEMD method, the amplitude of the white noise to be added is set as 0.2 and the ensemble number is set as 100. The KLD values between the original signal and IMF components are calculated and the first four components of IMF1, IMF2, IMF3 and IMF4 are selected to extract features. In the ensuing step, the effectiveness factors β*_i_* of all the 45 features computed by the DE method are shown in Figure 7, and the first eight features with the greatest values are listed in Table 1. Seen from the table, each feature represents different effectiveness factor, and the original signal and each component both have some relevant features. Selecting these features for the samples can enhance the diagnosis efficiency and accuracy. In addition, the cutting states of shearer (F1, F2, F3, F4 and F5) are marked as the levels of “1”, “2”, “3”, “4” and “5”, respectively.

### 5.2. Simulation Results of Proposed Method

**Figure 8:**
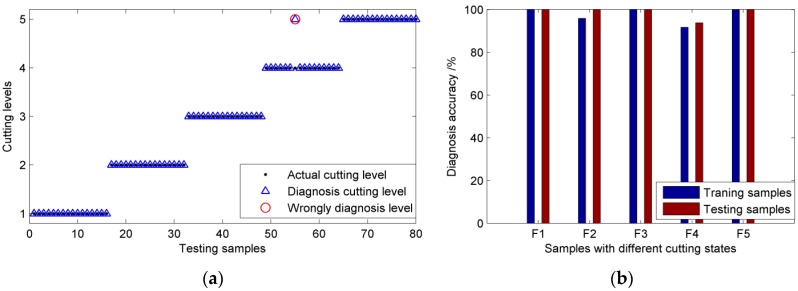
The diagnosis results based on proposed model (**a**) The testing results; (**b**) the diagnosis accuracies of different cutting states.

Based on the eight selected features, 200 samples for the proposed MFOA–PNN model can be generated. Sixty percent of the samples are used to optimize the smoothing parameters of PNN and the remaining samples are put into the trained PNN model to verify its classification performance. After some tries and simulations, the parameters of proposed method can be set as follows: *Maxgen* = 200, *sizepop* = 60, (*X_axis*, *Y_axis*) ⊂ [−1, 1], *n* = 8, *m* = 120, *N*_1_ = *N*_2_ = *N*_3_ = *N*_4_ = *N*_5_ = 24, *k* = 5. After the training phase, the PNN model with the optimal parameters can be obtained and the diagnosis results of the training samples and testing samples are illustrated in Figure 8.

As observed from Figure 8, only one sample is wrongly classified during the testing phase and the diagnosis accuracy can reach 98.75%. For the training samples, the diagnosis accuracies of different cutting states are 100%, 95.83%, 100%, 91.67% and 100%, respectively, and the overall diagnosis accuracy is 97.50%. The simulation results indicate that our proposed MFOA-PNN model possesses good generalization capability and is reliable to provide superior diagnosis ability for shearer cutting state.

### 5.3. Comparison with Other Methods

**Figure 9:**
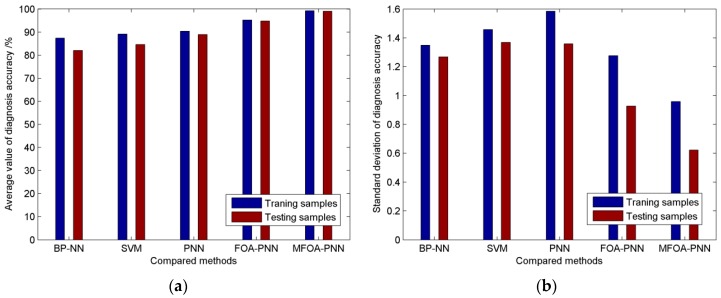
The diagnosis results based on different methods (**a**) The comparison of diagnosis accuracy; (**b**) the comparison of standard deviation of diagnosis accuracy.

To highlight the advantage of our proposed method over the conventional state diagnosis methods, it is compared with other four methods of FOA-PNN (the basic PNN with FOA), PNN (basic PNN), support vector machine (SVM), and back-propagation neural network (BP-NN). For the SVM-based state diagnosis method, the penalty parameter and kernel parameter are optimized by the K-fold cross validation method. The number of the input neurons of the BP-NN-based state diagnosis method is equal to the number of the selected features *u* and the number of the output neurons is set as the number of all the possible cutting types *k*. A single hidden layer structure is adopted and the number of neurons in the hidden layer is determined by an empirical formula $\sqrt{u+k}+1$. In addition, we set the same smoothing parameter for PNN based on the experience and other parameters of FOA-PNN and PNN can be set according to the proposed MFOA-PNN model. The training samples and testing samples are consistent with above simulation and the configurations of simulation environment for above methods are uniform. In order to verify the generalization ability, each method is trained and tested 20 times. The average value and standard deviation of the 20 training/testing accuracies are calculated. Finally, the compared results based on the five methods are illustrated in Figure 9.

It can be obviously observed from Figure 9 that the BP-NN- and SVM-based state diagnosis methods possess relatively poor training and testing accuracies compared with the other three methods, which are lower than 90%. Although the training (90.33%) and testing accuracy (88.94%) of PNN is a little higher, it is much lower than those of FOA-PNN (95.248% and 94.78%) and MFOA-PNN (99.22% and 99.04%). Furthermore, the standard deviation of proposed method is obviously smaller than other methods, which indicates that the diagnosis accuracy of MFO-PNN has smaller fluctuation and the proposed method performs better generalization ability. With the multi-swarm strategy for original FOA, the PNN with modified FOA can obtain better smoothing parameters and the MFOA-PNN model possesses excellent diagnosis accuracy among all the competing methods. Obviously the proposed MFOA-PNN can identify the cutting state of shearer with the vibration of rocker transmission part more accurately than the other four methods.

### 5.4. Further Studies for Different Parameter Settings

In the course of simulation, we notice that the number of selected features and the number of training samples are two critical parameters and have some influences on the diagnosis performance of some state diagnosis methods. Hence, the following subsection will present how the performance of above five methods may change with different parameter settings.

#### 5.4.1. The Number of Selected Features

**Figure 10:**
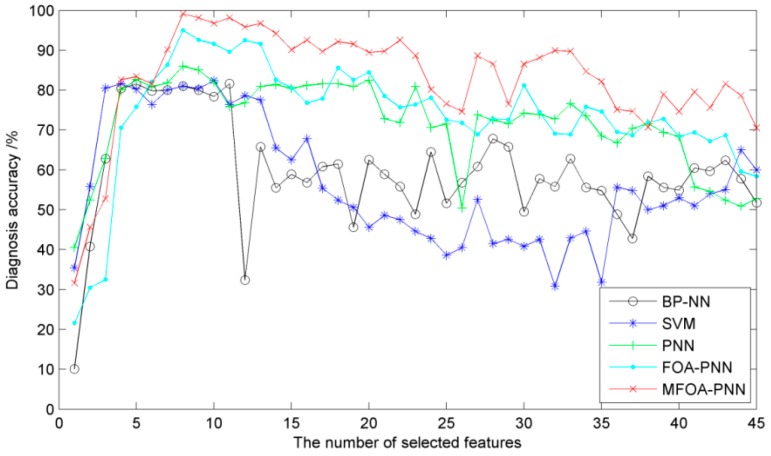
The diagnosis accuracies of the five methods with different numbers of selected features.

In order to measure the relation between the number of selected features and the diagnosis accuracy, the number of selected features for the vibration of rocker transmission part is set from 1 to 45 and the corresponding diagnosis accuracies based on different methods are compared. In this study, the number of training samples is set as 120 and the number of training samples is set as 80. The parameters of the diagnosis methods are chosen reasonably according to above simulation. These methods are trained and tested for 10 times and the average values are chosen as the diagnosis results. After the training phase, the diagnosis results of the testing samples for the BP-NN-based diagnosis method, SVM-based diagnosis method, PNN-based diagnosis method, FOA-PNN-based diagnosis method, and the proposed diagnosis method are plotted in Figure 10.

As can be seen in the graph, the diagnosis accuracies for all methods show an upward trend initially and then a downward trend with the increasing number of selected features. In detail, the BP-NN-based, SVM-based and PNN-based state diagnosis methods have an obvious fluctuation if the number of selected feature is greater than 10, whereas the mild oscillating phenomena occur for the FOA-PNN-based and MFOA-PNN-based state diagnosis methods. Furthermore, the proposed method always exhibits the highest accuracy than the other four methods for most selected features. The observation from Figure 10 illustrates that the proposed method, which directly considers the importance of selected features, possesses a superior performance with respect to the number of selected features than the competing methods.

However, using too many feature parameters to train the models will surely consume much time and reduce the computational efficiency for all the state diagnosis methods, although more selected features may increase the diagnosis accuracy. In order to balance the computational cost and diagnosis accuracy and according to the illustration in Figure 10, the first eight features are selected for our simulation studies.

#### 5.4.2. The Number of Training Samples

**Figure 11:**
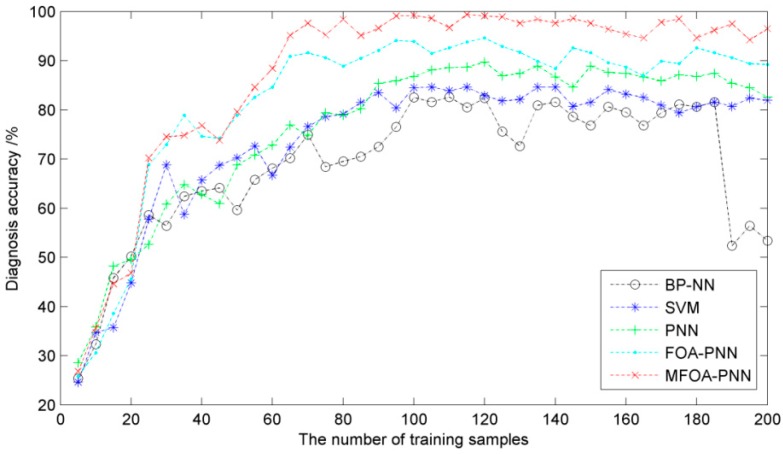
The diagnosis accuracies of the five methods with different numbers of training samples.

In this subsection, a comparative study is also carried out to reflect the influence of the number of training samples on the diagnosis accuracy. The first eight features with the greatest values of effectiveness factor are selected as the inputs of the five competing methods. The number of samples is added to 280 in order to obtain enough independent testing samples. The number of training samples changes from 5 to 200 and 80 testing samples are randomly selected from the residual samples for all diagnosis models. Meanwhile, the five methods also run 10 times for more accurate results. Finally, the diagnosis accuracies with respect to different numbers of training samples are illustrated in Figure 11.

It is observed from Figure 11 that the proposed method performs the best diagnosis accuracy than other methods when the number of the training samples is larger than 25. The diagnosis accuracies of the SVM-based, PNN-based, FOA-based and MFOA-based state diagnosis methods are stable and have faint fluctuations with the increase of training samples size. It is noteworthy that the accuracy curve of BP-NN-based state diagnosis method has a great decrease when the number of training samples becomes larger than 180. The reason for this phenomenon is that too many training samples may make the network over-trained and reduce the generalization ability of the network, leading to the difficulty in classifying state modes. Therefore, on the premise of ensuring diagnosis accuracy, the number of training samples for the state diagnosis methods is chosen as 120 in the simulation studies.

## 6. Conclusions and Future Work

The overall goal of the work presented in this paper is to provide a novel method for the diagnosis of shearer cutting state based on improved probabilistic neural network. The fruit fly optimization algorithm with a multi-swarm strategy is proposed for the adaptive choice of the smoothing parameters of the PNN. Then the Kullback-Leibler divergence is used to identify the false components from the EEMD, whereas the distance-based evaluation approach is employed to choose some effective features. Simulation experiments are provided and the comparisons with other four methods indicate that the proposed method possesses an exceptional performance on the state recognition of shearer and outperforms the competing methods.

We are confident that this study makes a significant contribution in the diagnosis of shearer cutting sate and will enable the production of high quality solutions for classification problems. However, this paper only consider the vibration of rocker transmission part to identify shearer cutting state and neglects the vibrations of other parts of shearer, such as travel unit and hydrocylinder. In the near future work, we will install some sensors on other key parts of shearer and acquire more useful vibration signals. Furthermore, some data fusion algorithms may be investigated to further improve the diagnosis accuracy and efficiency.

## Figures and Tables

**Table 1:** Eight selected features for the samples.

Feature ID	5	9	16	21
Feature type	*f*_5_ of signal	*f*_9_ of signal	*f*_7_ of IMF1	*f*_3_ of IMF2
β*_i_*	5.54	4.28	3.86	3.74
Feature ID	28	35	36	39
Feature type	*f*_1_ of IMF3	*f*_8_ of IMF3	*f*_9_ of IMF3	*f*_3_ of IMF4
β*_i_*	4.82	3.75	4.81	3.96
